# EUS-guided targeted epinephrine injection and immediate intraoperative bleeding during endoscopic mucosal resection: a retrospective cohort study (with video)

**DOI:** 10.3389/fmed.2026.1985945

**Published:** 2026-10-19

**Authors:** Wanting Wang, Aimei Rong, Chan Liao, Hongli Chen, Jiaqi Han, Qianping Liang

**Affiliations:** Department of Gastroenterology, Zhengzhou Central Hospital Affiliated to Zhengzhou University, Zhengzhou, Henan, China

**Keywords:** bleeding, endoscopic mucosal resection, endoscopic ultrasound, epinephrine, retrospective cohort study

## Abstract

**Objective:**

To investigate whether endoscopic ultrasound (EUS)-guided targeted epinephrine injection is associated with a lower rate of bleeding during endoscopic mucosal resection (EMR).

**Methods:**

This retrospective cohort study enrolled consecutive patients undergoing EMR for gastrointestinal lesions ≥1 cm with an EUS-detectable feeding vessel at Zhengzhou Central Hospital between January 2025 and March 2026. Patients were non-randomly assigned to EUS-guided targeted epinephrine injection (1:20,000) or EUS assessment followed by conventional submucosal saline injection. The primary outcome was immediate intraoperative bleeding; secondary outcomes included delayed bleeding, procedure time, hospital stay, *en bloc* resection rate, and adverse events. Multivariable logistic regression was performed, with Firth regression and propensity-score (PSM and IPTW) sensitivity analyses to address confounding.

**Results:**

Among 84 patients (injection group, *n* = 31; control group, *n* = 53), immediate intraoperative bleeding occurred in 0% versus 24.5% (*P* = 0.002). Two delayed bleeding cases (3.8%) occurred in the control group only. No between-group differences were observed in procedure time, hospital stay, or adverse events; the *en bloc* resection rate was 100% in both groups. EUS-guided targeted epinephrine injection was associated with reduced odds of intraoperative bleeding (OR = 0.112, 95% CI 0.028–0.443, *P* = 0.002), consistent across Firth regression, PSM, and IPTW analyses.

**Conclusion:**

EUS-guided targeted epinephrine injection was associated with lower immediate intraoperative bleeding during EMR without prolonging procedure time or hospital stay. These hypothesis-generating findings require confirmation in prospective randomized studies.

## 1 Introduction

Colorectal cancer and gastric cancer rank among the leading gastrointestinal malignancies worldwide in terms of both incidence and mortality (1, 2). With the advancement of minimally invasive endoscopic techniques, endoscopic mucosal resection (EMR) enables complete resection of early gastrointestinal lesions involving the mucosal layer and superficial submucosa, and has become the preferred minimally invasive treatment modality in clinical practice (3). Intraoperative and postoperative bleeding are the most common complications of EMR, with an overall incidence of approximately 3%–7.2% (4, 5). Lesions with a diameter of ≥1 cm, broad-based (sessile) polyps, and lesions with rich blood supply carry a higher risk of bleeding (6, 7). Once massive intraoperative bleeding occurs, it can cause blurred operative vision, prolonged procedure time, and even increase the risks of emergency surgery, blood transfusion, and readmission, thereby increasing the medical burden on patients (8).

To address the risk of bleeding, various prophylactic hemostatic strategies have been applied clinically, but each has limitations. Mechanical hemostasis (e.g., hemoclips) is effective but may cause tissue tearing due to excessive clipping or increase procedural costs (9); thermal coagulation (e.g., electrocoagulation) carries the risk of transmural injury or even perforation while sealing vessels (10); conventional blind submucosal epinephrine injection achieves hemostasis through vasoconstriction with a success rate of approximately 80% (11), but lacks real-time imaging guidance, making it difficult to precisely target feeding vessels, thus limiting its hemostatic efficacy.

In recent years, with the rapid development of endoscopic ultrasound (EUS) technology, EUS not only provides high-resolution real-time imaging of the gastrointestinal wall and surrounding structures, but also clearly identifies submucosal feeding vessels (12, 13). Its superior targeting capability has been confirmed in EUS-guided vascular interventions (e.g., precise devascularization of gastric varices) (14, 15). Recently, a case report has preliminarily explored the application of EUS-guided preemptive epinephrine injection in reducing bleeding risk during endoscopic submucosal dissection (16); however, controlled studies on intraoperative bleeding during EMR remain scarce. Therefore, this retrospective cohort study aimed to explore whether EUS-guided targeted epinephrine injection is associated with a lower rate of immediate intraoperative bleeding during EMR, and to generate hypotheses for prospective testing. Because treatment allocation was not randomized and lesions of the upper and lower gastrointestinal tract were pooled, the findings are reported as associations within a heterogeneous population and require validation in adequately powered prospective studies.

## 2 Materials and methods

### 2.1 General information

Consecutive patients with gastrointestinal lesions scheduled for EMR at the Department of Gastroenterology of our hospital between January 2025 and March 2026 were retrospectively enrolled.

**Inclusion criteria:** (1) endoscopically confirmed elevated gastrointestinal lesions or locally cancerous polyps with a diameter of ≥1 cm; (2) Clear vascular structures and blood flow signals at the lesion base detected by EUS, with a vessel diameter of ≥1 mm; (3) stable vital signs and basically normal cardiac, hepatic, and renal function; (4) complete clinical data and good compliance.

**Exclusion criteria:** (1) suspected deep infiltration or metastasis of the lesion; (2) severe cardiac, pulmonary, hepatic, or renal insufficiency; (3) severe mental disorders; (4) pregnancy or lactation; (5) allergy to epinephrine injection.

Patients were allocated non-randomly according to the intraoperative decision of the operating endoscopist. When colour Doppler imaging identified a clearly delineated feeding vessel at the lesion base, the endoscopist could decide intraoperatively, at his or her own discretion, to perform EUS-guided targeted injection of 1:20,000 epinephrine (injection group, *n* = 31); the remaining patients underwent EUS assessment of the lesion followed by conventional, non-targeted submucosal injection of saline without epinephrine (control group, *n* = 53). No standardized, pre-specified allocation protocol was in place, and allocation therefore reflected lesion vascularity and the operator's clinical experience; all cases were consecutively admitted patients during the same period at our centre. Because both groups underwent EUS assessment, the comparison made in this study is EUS-guided targeted epinephrine injection versus non-targeted saline injection. All operators had experience with ≥300 EMR procedures and ≥100 EUS puncture procedures. This study was approved by the Ethics Committee of Zhengzhou Central Hospital Affiliated to Zhengzhou University (Approval No.: ZXYY2026047). As a retrospective study, informed consent was waived.

### 2.2 Main equipment

Japanese PENTAX EG-3870UTK ultrasonic therapeutic endoscope; American COOK 19G EUS-specific disposable puncture needle; Olympus CV-290 electronic gastrointestinal endoscopy system, high-frequency electrosurgical generator; compatible hot biopsy forceps, metallic hemoclips, disposable snare, etc.

### 2.3 Procedure

#### 2.3.1 Injection group

After introducing 300–800 mL of distilled water through the working channel of the ultrasonic endoscope to enhance ultrasonic conduction, the origin layer and size of the lesion were evaluated using two-dimensional ultrasound and color Doppler mode, and the feeding vessels at the lesion base were precisely localized. Under real-time EUS guidance, a 19G puncture needle was used to inject 1:20,000 epinephrine saline at multiple points adjacent to the feeding vessels into the submucosa, with a total dose of 1.0–3.0 mL, individualized according to vascular distribution and mucosal lifting status. The criterion for adequate injection was uniform elevation of the mucosa around the lesion under EUS with significant weakening or disappearance of Doppler blood flow signals. After switching to a conventional gastrointestinal endoscope and confirming a positive lifting sign, standard snare EMR was performed to completely resect the lesion (Figure 1).

**Figure 1:**
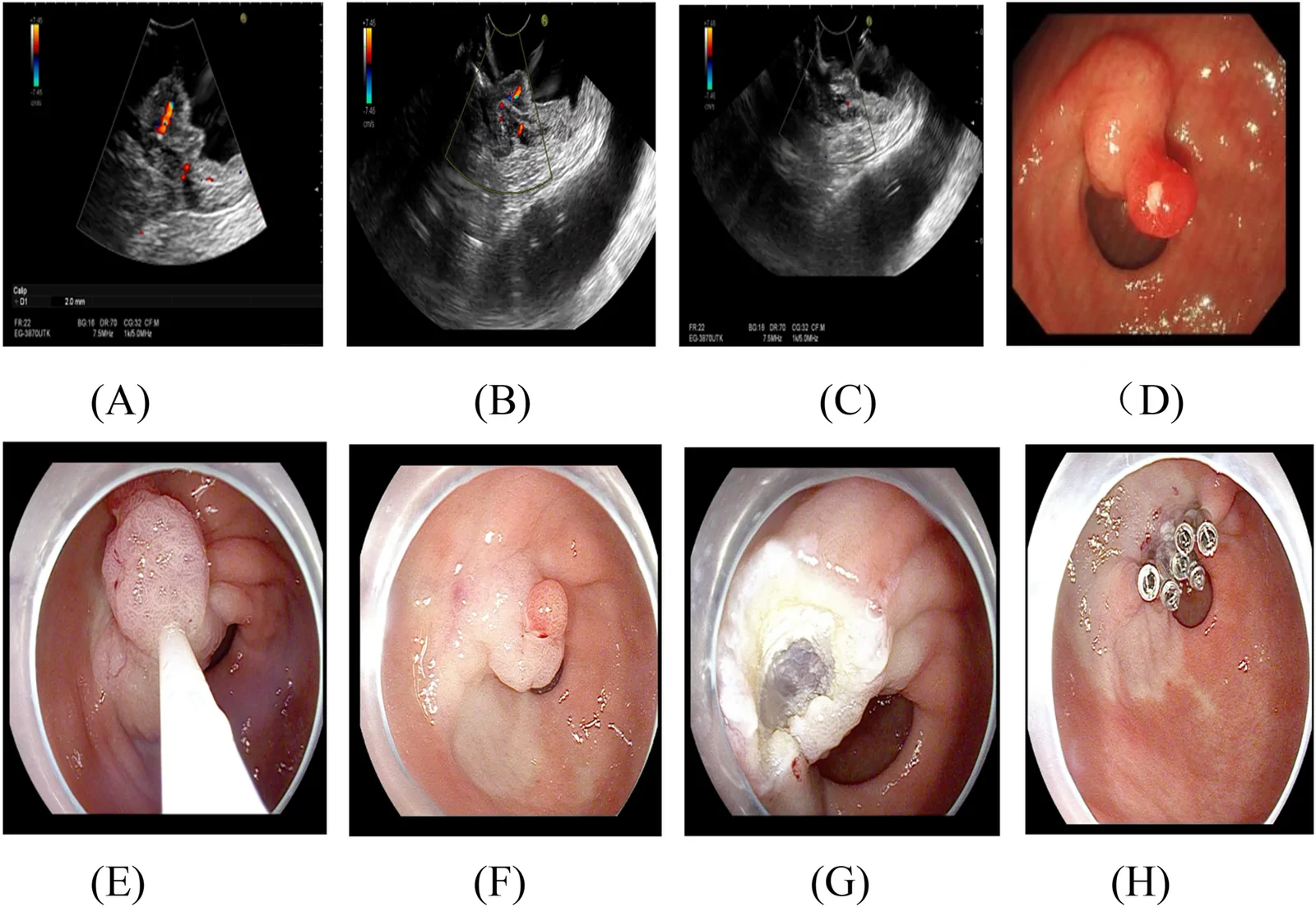
EUS-guided targeted epinephrine injection. **(A)** EUS showing a tubular feeding vessel (approximately 2 mm in diameter) penetrating from the lesion base. **(B)** Under EUS guidance, targeted injection of approximately 2 mL of 1:20,000 epinephrine saline adjacent to the feeding vessel using a disposable injection needle. **(C)** Disappearance of the Doppler blood-flow signal within the lesion after injection. **(D)** White-light endoscopic view of an elevated lesion measuring approximately 1.2 cm in the gastric antrum. **(E)** Targeted injection of epinephrine using a disposable injection needle under white-light endoscopy. **(F)** Blanching of the mucosa around the lesion after injection. **(G)** Post-EMR wound. **(H)** Prophylactic haemostasis using titanium clips.

#### 2.3.2 Control group (EUS assessment followed by conventional submucosal saline injection)

EUS was first performed in all patients to explore the lesion layer and the distribution of blood supply. After switching to a conventional gastrointestinal endoscope, saline solution alone (without epinephrine) was injected in a non-targeted manner into the submucosa at the lesion base to achieve mucosal lifting. After confirming a positive lifting sign, standard EMR resection was performed (Figure 2).

**Figure 2:**
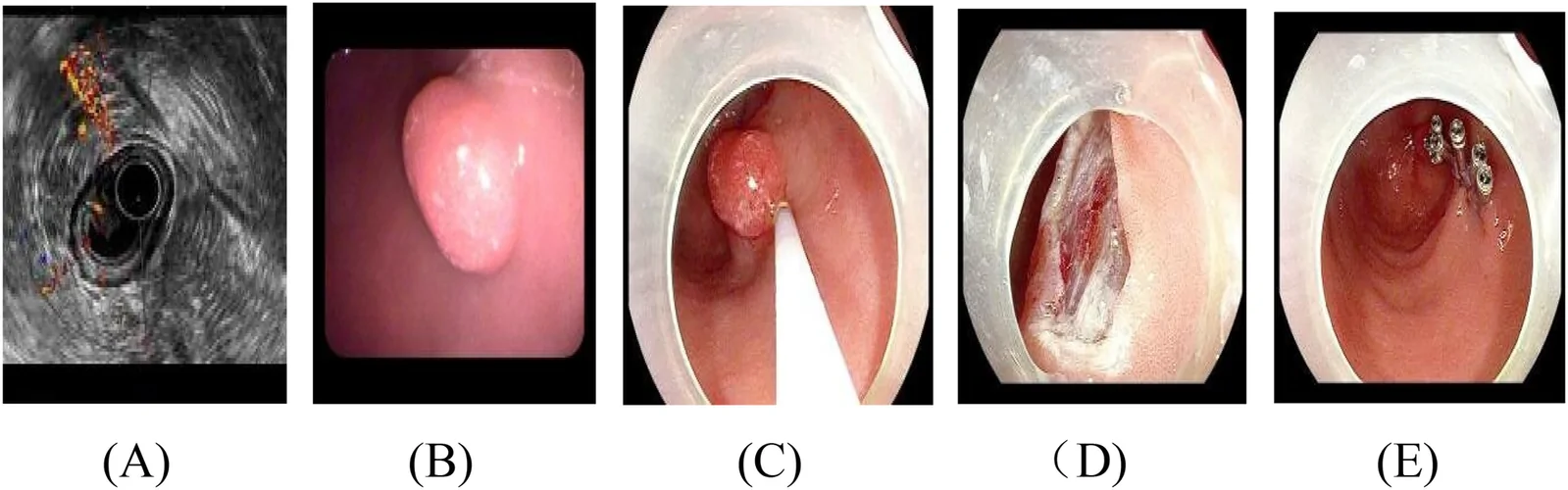
Procedure of endoscopic mucosal resection (EMR). **(A)** EUS showing a dendritic feeding vessel penetrating from the base of the lesion. **(B)** White-light endoscopic view of an elevated lesion measuring approximately 2 cm in the gastric antrum. **(C)** Submucosal injection of normal saline to lift the lesion. **(D)** Post-EMR wound. **(E)** Prophylactic haemostasis using titanium clips.

Both groups routinely underwent hemoclip closure of the wound surface after the procedure, and active bleeding was assisted by electrocoagulation hemostasis.

### 2.4 Histopathological evaluation

Resected specimens were immediately flattened and fixed in 10% neutral buffered formalin for 12–24 h, and continuously sectioned at a thickness of 2 mm. Two senior pathologists at our hospital independently reviewed the slides in a double-blind manner to assess the pathological type, size, and depth of invasion of the lesion; in case of disagreement, a third senior pathologist was consulted for arbitration.

### 2.5 Outcome measures

#### 2.5.1 Primary outcome

Immediate intraoperative bleeding: visible oozing or overt active bleeding from the wound surface during the procedure, as recorded in the endoscopy reports. For the present analysis, all events were re-reviewed and classified as oozing or active spurting bleeding, and the severity of bleeding was not routinely graded. Because prophylactic clip closure was performed routinely in all patients, the need for additional haemostatic intervention was not used in the definition of this endpoint.

#### 2.5.2 Secondary outcomes

(1) Delayed bleeding: first occurrence of melena or hematochezia within 14 days postoperatively, requiring blood transfusion or endoscopic intervention, or causing a decrease in hemoglobin ≥2 g/dL.
(2) En bloc resection: complete one-time resection of the entire lesion.
(3) Operation time: time from insertion of the ultrasonic endoscope to complete resection of the lesion.
(4) Endoscopy-related adverse events: graded according to the Adverse Events in Gastrointestinal Endoscopy (AGREE) classification system (21); severe cardiopulmonary adverse events related to epinephrine were judged according to the American Society for Gastrointestinal Endoscopy (ASGE) criteria (22).

### 2.6 Follow-up

All patients were uniformly followed up until 14 days postoperatively or April 2026, through outpatient review and telephone follow-up. No patients were lost to follow-up in this study.

### 2.7 Statistical analysis

SPSS 25.0 and R 4.5.3 software were used for statistical analysis. Continuous variables with normal distribution were expressed as mean ± standard deviation and compared using independent samples t-test; non-normally distributed continuous variables were expressed as median (Q1, Q3) and compared using Mann–Whitney U test. Categorical variables were expressed as number (percentage) [n (%)] and compared using chi-square test or Fisher's exact probability method. Multivariate logistic regression analysis was performed to identify independent influencing factors for intraoperative bleeding. Due to the limited sample size and small number of positive events, Firth penalized logistic regression was further adopted as a *post hoc* sensitivity analysis to address complete separation and the small number of events. Receiver operating characteristic (ROC) curve was plotted to evaluate the discrimination and calibration (Hosmer-Lemeshow goodness-of-fit test) of the model. To address confounding by indication arising from the non-randomized, physician-directed allocation, full propensity score matching and inverse probability of treatment weighting with stabilized weights were performed as sensitivity analyses, with all post-adjustment standardized mean differences < 0.10. *P* < 0.05 was considered statistically significant.

## 3 Results

### 3.1 Baseline characteristics

A total of 84 patients were enrolled in this study, including 45 males and 39 females. There were no statistically significant differences between the two groups in baseline indicators such as sex, age, smoking history, drinking history, underlying diseases (hypertension, diabetes, coronary heart disease, hyperlipidemia), antiplatelet drug use history, lesion size, or lesion location (all *P* > 0.05; Table 1); the distribution of Yamada classification differed significantly between groups (*P* = 0.009). This imbalance reflects the non-randomized, physician-directed allocation of treatment: lesions with a more prominent stalk or feeding vessel visible on EUS were more likely to be selected for targeted injection. Yamada classification was therefore included in the multivariable model and in the propensity models used for the sensitivity analyses. Detailed anatomical distribution by group, the distribution of lesion size as a continuous variable, and the histopathological distribution by group are reported in Supplementary Table S1.

**Table 1:** Comparison of baseline characteristics between the injection group and the control group in EMR patients.

Clinical Characteristics	Injection group (*n* = 31)	Control group (*n* = 53)	Statistic	*P*
Gender [n (%)]			*χ*^2^ = 0.399	0.528
Female	13 (41.9)	26 (49.1)		
Male	18 (58.1)	27 (50.9)		
Age (years) [n (%)]			χ^2^ = 0.001	0.970
<60	18 (58.1)	31 (58.5)		
≥60	13 (41.9)	22 (41.5)		
Smoking history [n (%)]	3 (9.7)	6 (11.3)		1.000^a^
Drinking history [n (%)]	4 (12.9)	4 (7.5)		0.459^a^
Hypertension [n (%)]	11 (35.5)	20 (37.7)	χ^2^ = 0.043	0.836
Diabetes [n (%)]	8 (25.8)	8 (15.1)	χ^2^ = 1.456	0.228
Coronary heart disease [n (%)]	6 (19.4)	6 (11.3)		0.345^a^
Hyperlipidemia [n (%)]	15 (48.4)	28 (52.8)	χ^2^ = 0.943	0.332
Antiplatelet drugs [n (%)]	8 (25.8)	9 (17.0)	χ^2^ = 0.944	0.331
Polyp size (cm) [n (%)]			χ^2^ = 1.194	0.275
<2	18 (58.1)	37 (69.8)		
≥2	13 (41.9)	16 (30.2)		
Polyp location [n (%)]			χ^2^ = 0.107	0.744
Upper gastrointestinal tract	17 (54.8)	31 (58.5)		
Lower gastrointestinal tract	14 (45.2)	22 (41.5)		
Yamada classification [n (%)]			Mann–Whitney U	0.009
Type I	6 (19.4)	18 (34.0)		
Type II	8 (25.8)	24 (45.3)		
Type III	10 (32.3)	5 (9.4)		
Type IV	7 (22.6)	6 (11.3)		

a Fisher's exact test.

b Mann–Whitney U test for the ordinal comparison of Yamada classification; all other categorical comparisons used the chi-square test.

Data are *n* (%).

### 3.2 Analysis of risk factors for intraoperative bleeding in EMR patients

#### 3.2.1 Univariate analysis

History of hypertension (*P* = 0.014) and EUS-targeted epinephrine injection (*P* = 0.004) were potentially associated with immediate intraoperative bleeding (Table 2). These univariable estimates are unadjusted and are reported as exploratory.

**Table 2:** Univariate analysis of factors associated with intraoperative bleeding in EMR patients.

Variable	*OR*	*95% CI*	*P*
Gender			
Female			
Male	0.523	0.209–1.310	0.166
Age(years)			
<60			
≥60	0.689	0.270–1.756	0.435
Smoking history	0.222	0.026–1.873	0.167
Drinking history	0.259	0.030–2.220	0.218
Hypertension	0.251	0.083–0.754	0.014
Diabetes	0.889	0.276–2.867	0.844
Coronary heart disease	1.000	0.274–3.656	1.000
Hyperlipidemia	0.970	0.367–2.563	0.951
Antiplatelet drugs	1.116	0.365–3.412	0.848
Polyp size (cm)			
< 2			
≥2	0.853	0.325–2.234	0.746
Polyp location			
Upper gastrointestinal tract			
Lower gastrointestinal tract	0.802	0.319–2.020	0.640
Yamada classification			
Type I			0.552
Type II	0.583	0.146–2.323	0.445
Type III	0.611	0.165–2.269	0.462
Type IV	0.292	0.055–1.550	0.148
Injection of epinephrine	0.179	0.055–0.583	0.004

Values are unadjusted (univariable) odds ratios with 95% confidence intervals;OR, odds ratio; CI, confidence interval.

#### 3.2.2 Multivariate logistic regression analysis

Immediate intraoperative bleeding occurred in 0 of 31 patients (0%) in the EUS-guided targeted epinephrine injection group and in 13 of 53 patients (24.5%) in the control group. With immediate intraoperative bleeding (no = 0, yes = 1) as the dependent variable, and history of hypertension (no = 0, yes = 1), EUS-targeted epinephrine injection (no = 0, yes = 1), and Yamada classification morphology (with Type I as the reference category and Types II–IV as dummy variables) as independent variables, multivariable logistic regression analysis was performed. These three covariates were selected *a priori* on clinical grounds and on univariate screening (*P* < 0.10), primarily to conserve degrees of freedom given the small number of events. After adjustment, EUS-guided targeted epinephrine injection was associated with lower odds of immediate intraoperative bleeding (OR = 0.112, 95% CI 0.028–0.443, *P* = 0.002); history of hypertension was likewise inversely associated with bleeding (OR = 0.199, 95%CI 0.060–0.656, *P* = 0.008), an unexpected finding that contradicts previous literature and is treated as hypothesis-generating. Yamada classification showed no independent association with intraoperative bleeding (*P* > 0.05). The model showed acceptable fit (Hosmer–Lemeshow *P* = 0.543); however, with only 13 events the events-per-variable ratio was approximately 2.6 with dummy variables for Yamada classification were included, so the adjusted estimates must be interpreted as exploratory and the odds ratios may be exaggerated (Table 3).

**Table 3:** Multivariate logistic analysis of factors associated with intraoperative bleeding in EMR patients.

Variable	*B*	*SE*	*Wald χ2*	*OR*	*95% CI*	*P*
Injection of epinephrine (yes vs no)	−2.190	0.702	9.736	0.112	0.028–0.443	0.002
Hypertension (yes vs no)	−1.615	0.609	7.035	0.199	0.060–0.656	0.008
Yamada classification (reference: Type I)			2.639			0.451
Type II vs Type I	−1.261	0.862	2.141	0.283	0.052–1.535	0.143
Type III vs Type I	−1.251	0.821	2.326	0.286	0.057–1.429	0.127
Type IV vs Type I	−1.084	1.003	1.167	0.338	0.047–2.417	0.280

The outcome variable was intraoperative bleeding during EMR (yes/no). Yamada classification was analyzed with Type I as the reference. The Hosmer-Lemeshow goodness-of-fit test yielded a *P* value of 0.543, indicating a good model fit.

### 3.3 Sensitivity and propensity-based analyses

In Firth penalized logistic regression, which was prespecified as the sensitivity analysis for separation and sparse events, the direction of the association was unchanged: EUS-targeted epinephrine injection (OR = 0.14, 95% CI 0.03–0.46, *P* = 0.0008) and history of hypertension (OR = 0.23, 95% CI 0.07–0.68, *P* = 0.0067) were inversely associated with intraoperative bleeding, and no association was found between Yamada classification and bleeding (Table 4). Because these estimates rest on only 13 events, they remain exploratory. To further mitigate confounding by indication, two propensity-based sensitivity analyses were performed with the same prespecified covariate set (age, sex, smoking, drinking, history of hypertension, diabetes, coronary heart disease, lesion size ≥2 cm, lesion location and Yamada classification). In full propensity score matching, all covariates were balanced after adjustment (all standardized mean differences < 0.10) and the association remained consistent (OR = 0.143, 95% CI 0.026–0.785); inverse probability of treatment weighting with stabilized weights gave a similar result (OR = 0.081, 95% CI 0.018–0.359). Hyperlipidaemia was not included in the propensity models because it was unassociated with bleeding in univariable analysis (*P* = 0.95). Although the direction and magnitude of the association were stable across all three adjustment strategies, residual confounding by indication—particularly from the intraoperative assessment of vascular density, which was not quantified in the medical records—cannot be excluded, and the propensity-adjusted estimates remain exploratory (Supplementary Table S2). ROC curve analysis of the multivariable model gave an area under the curve of 0.787 (95% CI 0.683–0.892, DeLong method), with an optimal model-predicted probability cut-off of 0.440 (sensitivity 71.4%, specificity 76.8%, Youden index 0.482); because a predictive model derived from 13 events cannot be regarded as validated, this analysis is presented as exploratory only (See Supplementary Figure S1).

**Table 4:** Firth logistic regression analysis of factors associated with intraoperative bleeding in EMR patients.

Model	Variable	*OR*	*95% CI*	*P*
Multivariate analysis				
	Hypertension (yes vs no)	0.23	0.07–0.68	0.0067
	Yamada classification (reference: Type I)			
	Type II vs Type I	1.00	0.30–3.37	0.9961
	Type III vs Type I	1.21	0.21–6.59	0.8274
	Type IV vs Type I	3.09	0.65–16.41	0.1561
	Injection of epinephrine (yes vs no)	0.14	0.03–0.46	0.0008

Firth penalized logistic regression was used as the prespecified sensitivity analysis to address separation and the small number of events; Yamada classification was entered with Type I as the reference. OR, odds ratio; CI, confidence interval.

### 3.4 Clinical efficacy and safety

The *en bloc* resection rate was 100% in both groups. Operation time and length of hospital stay did not differ significantly between the two groups (*P* > 0.05). Two delayed bleeding events occurred, both in the control group (2/53, 3.8%), and none in the injection group (0/31); these events are reported descriptively only, and no statistical comparison was performed because the study is grossly underpowered for this endpoint. Because prophylactic clip closure was performed routinely in all patients, the effect of epinephrine on delayed bleeding cannot be assessed independently. Two cases (6.5%) of endoscopy-related adverse events occurred in the injection group and five (9.4%) in the control group, including 2 cases of delayed bleeding (AGREE Grade II) and 3 cases of abnormal laboratory indicators requiring re-examination (AGREE Grade I) in the control group; all adverse events in the injection group were Grade I mild laboratory abnormalities, with no statistically significant difference in the incidence of adverse events between groups (*P* = 1.000). No perforation, severe epinephrine-related cardiopulmonary adverse reaction, or conversion to surgical procedures occurred in any patient; however, the absence of severe adverse events in 31 treated patients cannot establish safety (Table 5). An exploratory analysis of the interaction between lesion size and EUS-guided epinephrine injection was inconclusive (See Supplementary Table S3).

**Table 5:** Comparison of clinical efficacy.

Variables	Injection group (*n* = 31)	Control group (*n* = 53)	P
Delayed bleeding rate [n (%)]	0	2 (3.8)	-
En bloc resection rate [n (%)]	31 (100)	53 (100)	-
Operative time [min (M (Q1, Q3)]	25 (16, 45)	26 (18, 53)	0.871
Hospital stay [d (M (Q1, Q3)]	5 (4,9)	5 (4,8)	0.751
Endoscopy-related adverse even[n (%)]	2 (6.5%)	5 (9.4%)	1.000^a^

a Fisher's exact test.

b Mann–Whitney U test for operative time and length of hospital stay.

Data are *n* (%) or median (Q1, Q3).

## 4 Discussion

Intraoperative bleeding during EMR is a key complication affecting operative vision and safety. Existing EMR hemostatic strategies have certain limitations (11, 17). Conventional blind epinephrine injection lacks precision, whereas this study innovatively combines EUS guidance technology with prophylactic hemostasis. In this cohort, EUS-guided targeted epinephrine injection was associated with a lower observed rate of intraoperative bleeding (OR = 0.112) without increasing operation time, suggesting technical feasibility in experienced hands at a high-volume centre. Because allocation was physician-directed and non-randomized, this association is subject to confounding by indication; in addition, both groups underwent EUS assessment, so the comparison made here is EUS-guided targeted epinephrine injection versus non-targeted saline injection and no conclusion can be drawn about the value of EUS guidance itself. A two-by-two factorial randomized design (EUS-guided versus non-guided x epinephrine versus saline) would be required to separate these effects.

### 4.1 Mechanism of action

EUS combined with color Doppler enables visualization and localization of feeding vessels, addressing the shortcomings of conventional blind injection. Epinephrine exerts vasoconstrictive effects through activation of local vascular *α*-receptors (11, 18), reducing wound surface blood flow; meanwhile, the fluid cushion formed by submucosal injection produces a physical compression effect. These dual mechanisms synergistically reduce bleeding risk. This study showed no difference in operation time between the two groups, indicating that EUS-guided injection could be integrated into the routine EMR workflow at our centre; whether the same holds at lower-volume centres requires evaluation, and the technique should not yet be regarded as a standard of care.

### 4.2 Interpretation of the correlation between hypertension and intraoperative bleeding

History of hypertension appeared to be inversely associated with intraoperative bleeding, which contradicts the findings of Watabe et al. (19) and lacks a convincing biological explanation. The most plausible candidate explanations are residual confounding—for example, more intensive perioperative blood-pressure control and closer clinical surveillance of hypertensive patients in this cohort, together with the standard discontinuation of antiplatelet therapy before the procedure—statistical instability, given that the model was based on only 13 events, and chance. This observation must therefore be regarded as hypothesis-generating: it should not be interpreted as evidence that hypertension exerts a causal protective effect and should not be used to guide clinical management.

### 4.3 Lesion morphology and resection effect

Previous studies (20) have suggested that Yamada Type III lesions carry a relatively higher bleeding risk. In this study, Yamada classification showed no independent association with bleeding; this must not be interpreted as evidence that lesion morphology is unimportant, because the study had very limited power to detect such differences and because the baseline imbalance in morphology itself reflects the physician-directed allocation of lesions with a more prominent feeding vessel to the injection group. The 100% *en bloc* resection rate in both groups confirmed that this injection method does not interfere with EMR snare resection and can ensure complete lesion resection. In addition, 2 cases of delayed bleeding occurred in the control group (incidence 3.8%) and none in the injection group; the study generated a hypothesis that EUS-guided injection may reduce delayed bleeding, but larger studies are required. This result is basically consistent with previous reports on the incidence of delayed bleeding after EMR (4). Safety assessment showed no statistically significant difference in the incidence of endoscopy-related adverse events between the two groups, with no perforation, severe cardiovascular adverse reactions, or conversion to surgery; however, the absence of severe adverse events in 31 treated patients cannot establish safety, and the incidence of severe epinephrine-related adverse reactions therefore remains to be defined.

### 4.4 Cost and resource utilization

The proposed technique requires an ultrasonic therapeutic endoscope with colour Doppler capability, a 19G EUS puncture needle, and an endoscopist competent in EUS-guided puncture, all of which add equipment time and procedural complexity and therefore cost to each procedure. In this cohort, operation time was not increased, but whether the observed reduction in immediate bleeding justifies the additional resource utilization—particularly in settings with a low baseline bleeding risk—requires formal cost-effectiveness evaluation in prospective studies, and the threshold for adopting this technique will be setting-dependent.

### 4.5 Limitations

The limitations of this study include: (1) the design is single-centre, retrospective and non-randomized, with physician-directed allocation of treatment and consequently confounding by indication of uncertain direction; although propensity score matching and inverse probability of treatment weighting balanced the measured covariates, residual confounding by unmeasured intraoperative vascular density cannot be excluded; (2) only 13 immediate bleeding events occurred (events-per-variable ratio approximately 2.6), so the multivariable estimates are imprecise, may be overestimated and are exploratory;(3) lesions of the upper and lower gastrointestinal tract were pooled in the primary analysis, and because these lesions differ in morphology, histopathology, procedural technique and bleeding risk, the findings should be validated separately for upper and lower gastrointestinal lesions; (4) both groups underwent EUS assessment, so the independent contribution of EUS guidance cannot be determined from this design; (5) Because bleeding severity was not systematically documented in the source records, a severity-stratified analysis (oozing vs. spurting bleeding) could not be performed; moreover, since prophylactic clip closure was performed routinely in all patients and the primary endpoint did not require additional haemostatic intervention, clinically minor bleeding may have been captured, and the effect on delayed bleeding cannot be assessed independently; (6) all procedures were performed by experienced endoscopists (>300 EMR procedures and >100 EUS-puncture procedures) at a high-volume centre, so the results may not generalize to community hospitals or lower-volume centres and a learning-curve requirement should be anticipated; (7) the absence of severe adverse events in 31 treated patients cannot establish safety; and (8) follow-up was limited to 14 days and long-term recurrence data are lacking.

In conclusion, in this single-centre retrospective cohort, EUS-guided targeted epinephrine injection was associated with a lower observed rate of immediate intraoperative bleeding during EMR, without a measurable increase in operation time or length of hospital stay. Given the non-randomized, physician-directed allocation and the small number of events, these findings are hypothesis-generating, cannot establish efficacy or safety, and require confirmation in prospective randomized studies.

## Data Availability

The raw data supporting the conclusions of this article will be made available by the authors, without undue reservation.
